# A retrospective study to evaluate the effect of preoperative hormonal therapy on continence recovery

**DOI:** 10.3389/fonc.2022.1059410

**Published:** 2023-01-13

**Authors:** Yuwen Wang, Shun Zhang, Haifeng Huang, Xuefeng Qiu, Yao Fu, Xiaoyu Lyu, Linfeng Xu, Junlong Zhuang, Hongqian Guo

**Affiliations:** ^1^ Department of Urology, Affiliated Drum Tower Hospital, Medical School of Nanjing University, Nanjing, China; ^2^ Medical School of Southeast University Nanjing Drum Tower Hospital, Nanjing, China; ^3^ Institute of Urology, Nanjing University, Nanjing, China; ^4^ Department of Pathology, Affiliated Drum Tower Hospital, Medical School of Nanjing University, Nanjing, China

**Keywords:** hormonal therapy, continence, prostatectomy, oligometastatic prostate cancer, locally advanced prostate cancer

## Abstract

**Objective:**

To evaluate whether different preoperative hormonal therapy options affect postoperative continence and to identify risk/protective factors for continence recovery.

**Methods:**

This is a retrospective analysis of several clinical trials (NCT04356430, NCT04869371, NCT04992026 and NCT05406999). Data from patients treated with hormonal therapy followed by RARP were collected and analyzed. Continence was defined as 0 pad/day or one safety pad.

**Results:**

The study included 230 patients with adequate information. The median time to continence recovery is 8 weeks. A total of 216 (93.9%) participants recovered to urinary continence within 12 months after surgery. 21 (9.1%) participants achieved immediate continence. 69, 85, 27 and 14 participants restored continence at 1 month, 1-3 month, 3-6 month, 6-12 month, accounting for 30.0%, 40.0%, 11.7% and 6.1% accordingly. No difference in continence recovery was found among different preoperative hormonal treatment options (*p*=0.821). Cox regression showed that membranous urethral length (MUL) was the only independent factor influencing urinary continence recovery either in the univariate analysis (OR=1.13, 95%CI: 1.04-1.22, p=0.002) or in the multivariate analysis (OR=1.12, 95%CI: 1.04-1.20, p=0.002). Different preoperative treatment options were not associated with urinary recovery. More advanced preoperative T stage (OR=0.46, 95%CI: 0.24-0.85, p=0.014) delayed the recovery of immediate continence. MUL was associated with continence restoring at 1 month (OR=1.20, 95%CI: 1.03-1.39, p=0.017), 3 month (OR=1.27, 95%CI: 1.07-1.51, p=0.006), 6 month (OR=1.34, 95%CI: 1.07-1.67, p=0.011) and 12 month (OR=1.36, 95%CI: 1.01-1.84, p=0.044).

**Conclusion:**

There is no difference in postoperative continence recovery among ADT, ADT+Docetaxel and ADT+Abiraterone preoperative treatment options. More advanced T stage indicated poor immediate continence recovery. Longer membranous urethral length was a promotional factor for both short-time and long-time continence recovery.

## 1 Introduction

Urinary incontinence following robot-assisted radical prostatectomy (RARP) is a significant and perhaps under-reported consequence that substantially decreases quality of life (QOL) (1). Besides oncological outcomes after RARP in prostate cancer (PCa) patients, functional results have become another focus of attention (2). Emerging surgical techniques including bladder neck preservation, selective dorsal venous complex, nerve-sparing technique, and posterior musculofascial reconstruction as well as anterior restoration of the pelvis space were suggested by surgeons and showed promising improvements in restoring continence (3–7). However, there was 8% to 11% of patients still suffering from incontinence one year after RARP according to meta-analysis when the safety pad definition was used (7).

Androgen deprivation therapy (ADT) has been the standard of care for over 50 years for metastatic PCa (8). Combined hormonal therapy such as chemo-hormonal treatment and addition of new hormonal treatments (abiraterone, apalutamide, enzalutamide) was suggested by the STAMPEDE, CHAARTED and LATITUDE trials (9–11). Though lack of robust evidence, cytoreductive radical prostatectomy (CRP) in combination with hormonal therapy was hypothetically expected to reduce tumor burden, induce immune modulation and improved response to secondary treatment (12). A number of ongoing clinical trials might provide future evidence for the therapeutic effect of CRP (13–15).

The effects of neoadjuvant hormonal therapy (NHT) for high risk PCa have been a popular concern for many years, though the oncological results remained controversial. As for functional aspects, researchers found that neoadjuvant hormonal therapy resulted in immediate impairment of vitality and sexual quality of life (16). However, few studies looked into the effect on urinary continence. On the other hand, most of studies on postoperative continence excluded those previously treated with hormonal therapy due to potential bias, leaving this topic undiscovered (17–19).

Several clinical trials at our center focusing on hormonal therapy followed by RARP for localized, locally advanced and metastatic PCa with hundreds of participants were under way, which happened to be suitable for the continence research. The purpose of this study was to evaluate whether different preoperative hormonal therapy options affect postoperative continence and to identify risk/protective factors for incontinence.

## 2 Methods and materials

### 2.1 Patient selection

From December 2018 to May 2021, a total of 235 consecutive PCa patients from several phase 2 clinical trials (*ClinicalTrials.gov:* NCT04356430, NCT04869371, NCT04992026 and NCT05406999), who were treated with hormonal therapy followed by RARP were retrospectively collected. 230 well documented patients out of 235 patients were then included in the analysis. The study was approved by the Medical Ethics Committee of Nanjing Drum Tower Hospital, China.

According to the inclusion and exclusion criteria of the trials, patients with high risk localized (T1-2, N0, M0), locally advanced (T3-4, N0-1, M0) or oligometastatic PCa (no more than 5 metastatic lesions, no visceral metastasis) were all included in this analysis. All patients were diagnosed PCa with biopsy and went through careful examination including 1) Transrectal prostate ultrasonography; 2) prostate multi-parameter magnetic resonance imaging (mpMRI); 3) ECT plus CT scan of the whole abdomen or ^68^Ga-labeled molecular imaging with PET-targeted prostate-specific membrane antigen (^68^Ga-PSMA PET). Ultrasonography and mpMRI of prostate were carried out again after preoperative hormonal therapy to re-evaluated tumor conditions before surgery.

### 2.2 Preoperative therapy

The duration of preoperative hormonal therapy is 6 months. Regarding medication modality, it can be divided into the following three cases 1) ADT, hypodermic injection of luteinizing hormone-releasing hormone analog (LHRHa) every 12 weeks; 2) ADT+Docetaxel, ADT with additional intravenous administration of docetaxel 75 mg/m^2^ body surface area every 3 weeks for 6 cycles; 3) ADT+Abiraterone, ADT with additional daily 1000 mg of abiraterone acetate orally.

### 2.3 Surgical technique

The surgical technique was accomplished using da Vinci Surgical System. Robot-assisted radical prostatectomy (RARP) plus enlarged pelvic lymph node dissection (ePLND) within 2 weeks after the end of the therapy were performed by the same experienced surgeon (Dr. HG). Conventional robot-assisted radical prostatectomy (C-RARP), also known as anterior approach or Retzius-sparing robot-assisted radical prostatectomy (RS-RARP), also known as posterior approach was carefully chosen based on tumor conditions (tumor location, tumor stage and tumor lesion volume) and physical conditions (age and systematic complications). Other techniques, which could possibly improve continence were also applied including preserving maximal urethral length, dorsal venous complex ligation (DVCL) and posterior reconstruction (PR). Nerve sparing was not applied due to oncologic consideration.

### 2.4 Follow up and continence evaluation

Patients were discharged 4-6 days after surgery and the urinary catheter was removed on the 14^th^ postoperative day. All patients were encouraged to practice Kegels exercise. The follow-up was continued until urinary continence, which was defined as 0 pad/day or one safety pad. Immediate continence was defined as continence within 7 days after the removal of catheter.

### 2.5 Data collection

**Figure 1:**
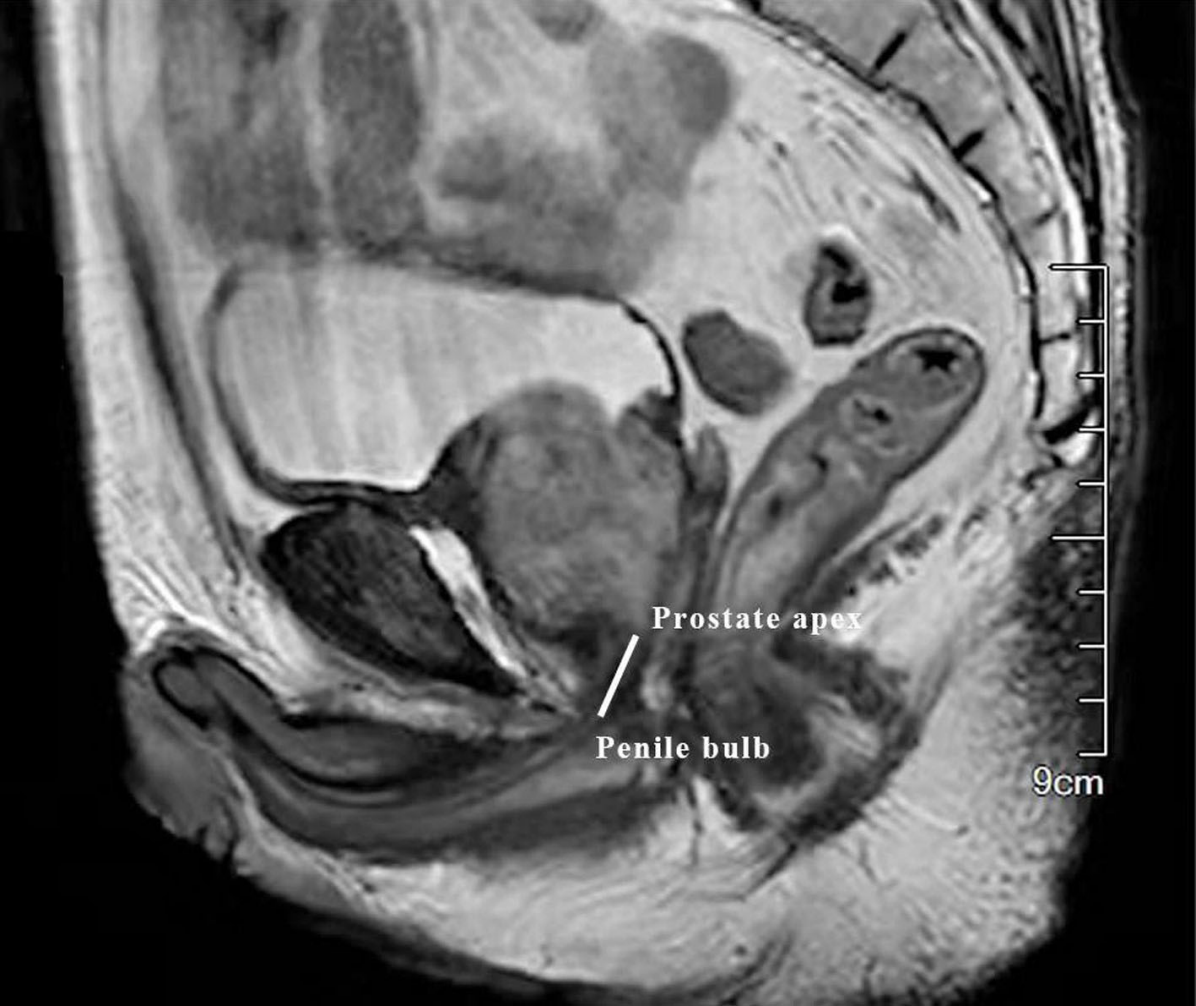
The measurement of membranous urethral length (MUL). The membranous urethral length (MUL) was measured from the prostate apex to penile bulb on the preoperative sagittal plane of T2-weighted MRI.

Patients’ data were extracted from their medical records. To be specific, basic information (age, BMI), information at initial diagnosis (PSA, TNM stage, biopsy Gleason, apex invasion or not), preoperative characteristics (membranous urethral length, PSA, prostate volume), preoperative therapy, surgery approach, post-surgery information (pathological T and N stage, surgical margin, post-surgery treatment) were collected. The membranous urethral length (MUL) (**Figure 1**) was measured on mpMRI.

### 2.6 Statistical analysis

Statistical analysis was realized by SPSS version 22.0 (IBM SPSS, Chicago, IL, USA). Continuous variables were presented as mean ± standard deviation (SD) or median and interquartile range (IQR). Categorical variables were reported as absolute frequency (percentage). One-way ANOVA (Analysis of Variance) or Kruskal-Wallis test was used to compare continuous variables between groups while Chi-square test or Fisher’s exact test was used to compare categorical variables between groups. Cox regression as well as logistic regression analysis were then sequentially applied for univariate and multivariate analysis. Age, BMI, initial T stage, apex invasion, preoperative PSA, preoperative volume, membranous urethral length, preoperative T, preoperative therapy, surgery approach and post-surgery ADT were included as evaluated variables. P < 0.05 was considered statistically significant.

## 3 Results

Data from 235 consecutive PCa patients who received preoperative hormonal therapy followed by RARP were collected from December 2018 to May 2021. 5 patients were then excluded due to refusal of phone interview. The average age was 69.00 ± 6.90 years and the average BMI was 24.50 ± 2.94 kg/m^2^. Participants’ basic characteristics were summarized in **Table 1**.

**Table 1:** Summary of Characteristics.

	Total	ADT (N=45)	ADT+Docetaxel (N=50)	ADT+Abiraterone (N=135)	*p*
Basic characteristic					
Age, y, Mean ± SD	69.00 ± 6.90	70.58 ± 5.89	68.26 ± 7.28	68.75 ± 6.59	0.237
BMI, kg/m^2,^ Mean ± SD	24.50 ± 2.94	23.76 ± 3.01	24.40 ± 2.88	24.78 ± 3.13	0.358
Characteristic at initial diagnosis					
PSA, ng/ml, IQR	40.17 (18.94-75.13)	40.9 (13.70-56.99)	57.26 (19.76-100)	38.80 (19.46-69.76)	0.103
T stage, n(%)					0.756
T2	47 (20.4)	10 (22.2)	10 (20.0)	27 (20.0)	
T3a	54 (23.5)	9 (20.0)	14 (28.0)	31 (23.0)	
T3b	88 (38.3)	16 (35.6)	21 (42.0)	51 (37.8)	
T4	41 (17.8)	10 (22.2)	5 (10.0)	26 (19.3)	
N stage, n(%)					<0.001**
N0	149 (64.8)	40 (88.9)	50 (100.0)	59 (43.7)	
N1	81 (35.2)	5 (11.1)	0 (0.0)	76 (56.3)	
M stage, n(%)					<0.001**
M0	204 (88.7)	45 (100.0)	50 (100.0)	109 (80.7)	
M1	26 (11.3)	0 (0.0)	0 (0.0)	26 (19.3)	
ISUP, n(%)					0.845
1	40 (17.4)	10 (22.2)	10 (20.0)	20 (14.8)	
2	5 (2.2)	1 (2.2)	2 (4.0)	2 (1.5)	
3	53 (23.0)	10 (22.2)	13 (26.0)	30 (22.2)	
4	106 (46.1)	19 (42.2)	20 (40.0)	67 (49.6)	
5	26 (11.3)	5 (11.1)	5 (10.0)	16 (11.9)	
Apex invasion, n(%)					0.013*
Yes	103 (44.8)	23 (51.1)	30 (60.0)	50 (37.0)	
No	127 (55.2)	22 (48.9)	20 (40.0)	85 (63.0)	
Preoperative characteristic					
PSA, ng/ml, IQR	0.05 (0.01-0.19)	0.12 (0.04-0.41)	0.14 (0.03-0.53)	0.03 (0.01-0.10)	<0.001**
Prostate volume, ml, Mean ± SD	17.50 (13.60-22.73)	18.90 (14.50-25.25)	18.00 (14.60-23.05)	18.00 (14.63-23.05)	0.286
Membranous urethral length, mm, Mean ± SD	15.19 ± 1.87	14.77 ± 2.01	15.34 ± 2.03	15.28 ± 1.75	0.243
T stage, n(%)					0.170
T2	91 (39.6)	20 (44.4)	12 (24.0)	59 (43.7)	
T3a	61 (26.5)	14 (37.1)	16 (32.0)	31 (23.0)	
T3b	65 (28.3)	8 (17.8)	19 (38.0)	38 (28.1)	
T4	13 (5.7)	3 (6.7)	3 (6.0)	7 (5.2)	
Surgery					0.768
Anterior approach RARP	149 (64.8)	30 (66.7)	34 (68.0)	85 (63.0)	
Posterior approach RARP	81 (35.2)	15 (33.3)	16 (32.0)	50 (37.0)	
Post-surgery information					
Pathological T					0.302
T0	12 (5.2)	1 (2.2)	3 (6.0)	8 (5.9)	
T2	103 (44.8)	19 (42.2)	20 (40.0)	64 (47.4)	
T3a	61 (26.5)	18 (40.0)	13 (26.0)	30 (22.2)	
T3b	54 (23.5)	7 (15.6)	14 (28.0)	33 (24.4)	
Margin					0.918
Positive	46 (20.0)	9 (20.0)	9 (18.0)	28 (20.7)	
Negative	184 (80.0)	36 (80.0)	41 (82.0)	107 (79.3)	
Pathological N stage					0.352
N0	177 (77.0)	38 (84.4)	39 (78.0)	100 (74.1)	
N1	53 (23.0)	7 (15.6)	11 (22.0)	35 (25.9)	
Post-surgery ADT, n (%)					0.391
Yes	47 (20.4)	6 (13.3)	12 (24.0)	29 (21.5)	
No	183 (79.6)	39 (86.7)	38 (76.0)	106 (78.5)	

**p < 0.01

BMI, Body mass index; PSA, Prostate specific antigen; ISUP, International Society of Urological Pathology; RARP, Robot-assisted radical prostatectomy; ADT, Androgen deprivation therapy.

**Figure 2:**
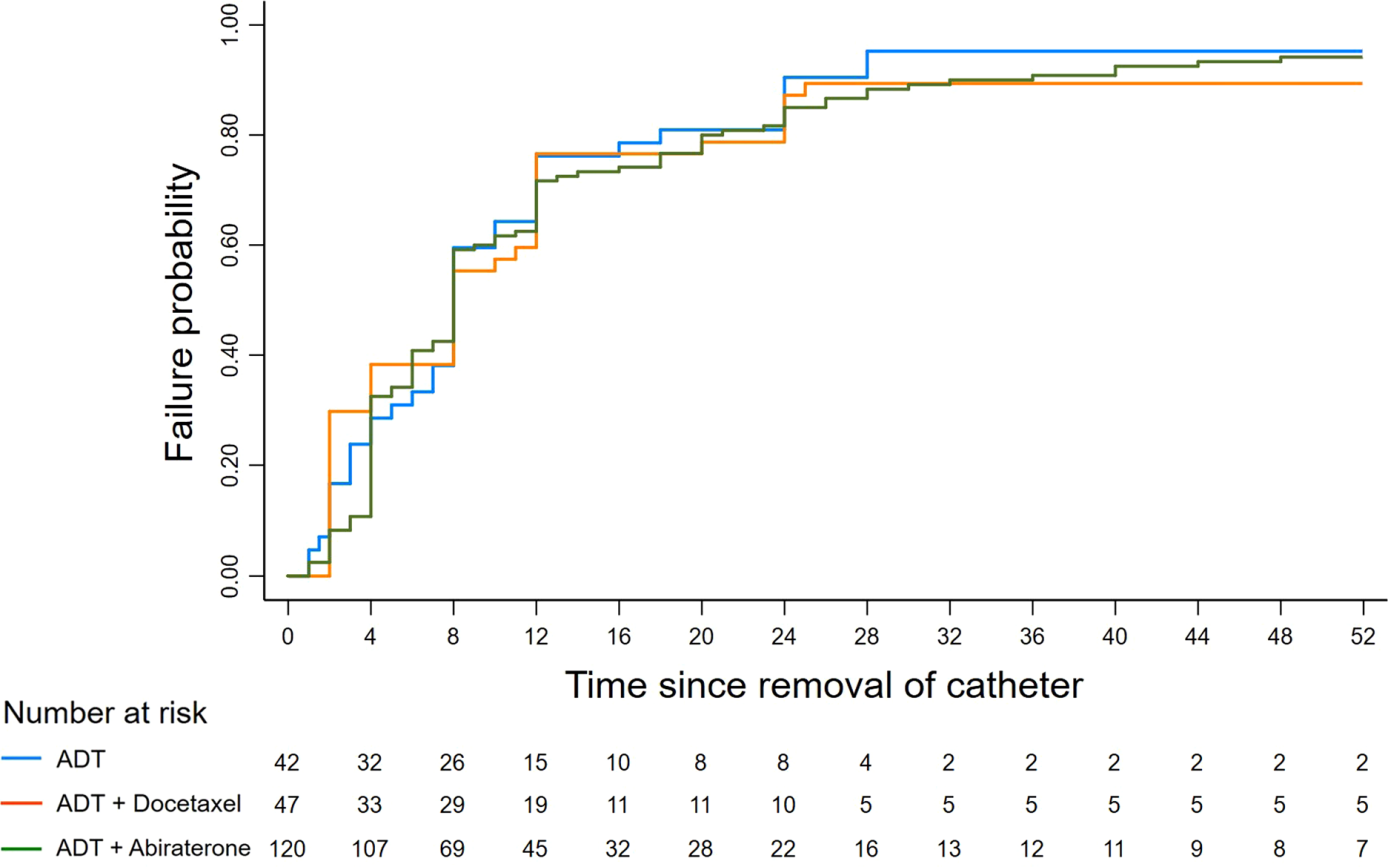
Kaplan-Meier failure graph of continence recovery. The Kaplan-Meier failure graph demonstrates continence results under different preoperative treatment conditions. No difference was found among the ADT, ADT + Docetaxel and ADT + Abiraterone groups (Log rank *p*=0.821).

The median time to continence recovery is 8 weeks. A total of 216 (93.9%) participants recovered to urinary continence within 12 months after surgery, leaving 14 (6.1%) not recovered at one year follow-up. 21 (9.1%) participants achieved immediate continence. 69, 85, 27 and 14 participants restored continence at 1 month, 1-3 month, 3-6 month, 6-12 month, accounting for 30.0%, 40.0%, 11.7% and 6.1% accordingly. More detailed information regarding time to continence recovery were illustrated in **Table 2**. No difference in continence recovery was found among different preoperative treatment options (*p*=0.821), as visualized in **Figure 2**.

**Table 2:** Time to urinary continence.

	Absolute Number	Accumulated Number	Absolute Percentage (%)	Accumulated Percentage (%)
Immediate	21	21	9.1	9.1
1 month	69	90	30.0	39.1
3 month	85	175	40.0	76.1
6 month	27	202	11.7	87.8
12 month	14	216	6.1	93.9
>12month	14	230	6.1	100

After including evaluated variables in the Cox regression analysis (**Table 3**), it showed that membranous urethral length (MUL) was the only independent factor influencing recovery time of urinary continence either in the univariate analysis (OR=1.13, 95%CI: 1.04-1.22, p=0.002) or in the multivariate analysis (OR=1.12, 95%CI: 1.04-1.20, p=0.002). It turned out that different preoperative treatment options (ADT, ADT+Docetaxel and ADT+Abiraterone) were not associated with urinary recovery.

**Table 3:** Cox regression model for urinary continence.

Factor	Univariate		Multivariate	
	OR (95% CI)	*p*	OR (95% CI)	*p*
Age	0.99 (0.97-1.01)	0.240	–	–
BMI	1.00 (0.95-1.06)	0.963	–	–
Initial T	0.88 (0.74-1.06)	0.184	–	–
Apex invasion	1.03 (0.78-1.36)	0.820	–	–
Preoperative PSA	0.99 (0.95-1.03)	0.632	–	–
Preoperative volume	1.00 (0.98-1.02)	0.844	–	–
Membranous urethral length	1.13 (1.04-1.22)	0.002^**^	1.12 (1.04-1.20)	0.002^**^
Preoperative T	1.05 (0.87-1.27)	0.590	–	–
Preoperative therapy				–
ADT + Docetaxel vs ADT	0.81 (0.52-1.26)	0.352	–	–
ADT + Abiraterone vs ADT	0.95 (0.66-1.38)	0.794	–	–
Surgery approach	1.04 (0.78-1.39)	0.781	–	–
Post-surgery ADT	0.79 (0.55-1.15)	0.781	0.76 (0.54-1.06)	0.109

**p < 0.01.

BMI, Body mass index; PSA, Prostate specific antigen; ADT, Androgen deprivation therapy; OR, Odds ratio; CI, Confidence Interval.

Deeper digging into the potential risk or protective factors for urinary continence at different time were then carried out using logistic regression (**Supplementary Table 1**). As for immediate continence, only preoperative T stage was correlated (OR=0.46, 95%CI: 0.24-0.85, p=0.014). MUL was associated with continence recovery at 1 month (OR=1.20, 95%CI: 1.03-1.39, p=0.017), 3 month (OR=1.27, 95%CI: 1.07-1.51, p=0.006), 6 month (OR=1.34, 95%CI: 1.07-1.67, p=0.011) and 12 month (OR=1.36, 95%CI: 1.01-1.84, p=0.044).

A portion of the patients continued to receive ADT shortly after surgery (**Supplementary Table 2**). To further investigate the potential role of postoperative hormonal therapy on continence recovery, subgroup analysis was carried out. Results showed that postoperative ADT delayed continence recovery in PCa patients previously treated with ADT+Docetaxel (*p*=0.005) while not in patients previously treated with ADT alone (*p*=0.232) or ADT+Abiraterone (*p*=0.805) (**Supplementary Figure 1**).

## 4 Discussion

The results of the study showed that 9.1% and 93.9% of patients restored continence immediately and 1 year after removal of catheter. No difference was found in postoperative continence recovery among ADT, ADT+Docetaxel and ADT+Abiraterone preoperative treatment options. More advanced T stage increased the risk of immediate incontinence and longer membranous urethral length (MUL) promoted continence at 1, 3, 6 and 12 month.

Radical prostatectomy leads to anatomical impairment to urethral sphincter complex, its surrounding tissue and innervation, which cause incontinence (20, 21). In addition, extensive dissection, neurovascular bundle (NVB) damage and postoperative fibrosis also impose a negative effect on post-prostatectomy continence recovery (20). We found that preoperative T was the only factor that affect immediate continence. Even after 6-month preoperative hormonal therapy, more than 60% of the participants were deemed to have extraprostatic invasion. The adhesions of adjacent tissues and loss of clear boundaries caused by advanced tumor stage forced the surgeon to extend the extrafascial dissection plane in order to reduce the rate of positive surgical margin (PSM). It’s possible that extensive dissection posed a negative impact on continence.

Current evidence exhibited significant advantage of RS-RARP over C-RARP in terms of immediate continence recovery while not in long-term continence recovery (22–24). However, most of these researches have explicitly ruled out patients previously treated with preoperative hormonal treatment. Our results showed that surgical approaches (RS-RARP or C-RARP) did not affect immediate, short-time, median-time or long-time continence recovery. Though the matter of continence is influenced by patients’ preoperative characteristics, surgeon experience, surgical techniques and methodological aspects such as continence definitions, tools used for data collection, and different follow-up intervals (7), it might be reasonable to suggest that RS-RARP is not superior as expected in this specific setting. One possible explanation is that the significant shrinkage of tumor volume after neoadjuvant therapy leads to the increase in maximum urethra length permissible to be retained. The increase in functional urethra length might eliminate the impact caused by different surgery approaches (RS-RARP or C-RARP), which is consistent with our conclusion that MUL is the most important factor influencing postoperative continence for patients with preoperative hormonal therapy. The RS-RARP would be less preferable if taking cancer control into account, as it was reported by several studies to increase the risk of PSM (22, 23).

Preoperative membranous urethral length (MUL) was shown to be a crucial factor influencing continence recovery, which is consistent with former researches (25, 26). The combined and coordinated function of smooth muscle fibers and the surrounding rhabdosphincter, which are two main components of membranous urethra contributes to maintaining the urethral closure pressure (27, 28). The longer MUL provide better urethral pressure profile, thus prompting continence recovery.

Though our research demonstrated that different preoperative treatment options did not make a difference on continence recovery, it did show that sustained postoperative hormonal therapy might impair continence recovery. The impact of occurent or previous hormonal therapy on postoperative continence still needs to be further discussed.

There are several limitations of this study. Firstly, this is a single center, single surgeon retrospective study. The learning curve and retrospective nature might undermine the general implication of the study. Secondly, the varying and complicated patients’ characteristics might cause potential bias. Despite the limitations, this study provided evidence of the impact of different preoperative pharmacotherapy on postoperative urinary continence, which was scarcely ever mentioned in existing research.

## 5 Conclusion

In the study, 93.9% participants recovered to urinary continence within 12 months after surgery. There is no difference in postoperative continence recovery among ADT, ADT+Docetaxel and ADT+Abiraterone preoperative treatment options. More advanced T stage indicated poor immediate continence recovery. Longer membranous urethral length was a promotional factor for short-time and long-time continence recovery.

## Data availability statement

The raw data supporting the conclusions of this article will be made available by the authors, without undue reservation.

## Ethics statement

The studies involving human participants were reviewed and approved by Medical Ethics Committee of Nanjing Drum Tower Hospital, China. Written informed consent for participation was not required for this study in accordance with the national legislation and the institutional requirements. Written informed consent was not obtained from the individual(s) for the publication of any potentially identifiable images or data included in this article.

## Author contributions

JZ and YW designed and planned the study protocol. HG and LX supervised the work. YW, SZ and XL collected patients’ clinical data while YF provided pathology reports. YW and HH performed the analysis, drafted the manuscript and designed the figures. JZ and XQ aided in interpreting the results and worked on the manuscript. All authors contributed to the article and approved the submitted version.
