# Supplementary material for: A retrospective study to evaluate the effect of preoperative hormonal therapy on continence recovery
Source: Front Oncol. 2023 Jan 13;12:1059410. doi: 10.3389/fonc.2022.1059410 (PMC9880985; doi:10.3389/fonc.2022.1059410)

Supplementary Figure 1. The Kaplan-Meier failure graph of postoperative ADT on continence recovery.

The Kaplan-Meier failure graph demonstrates subgroup analysis of the effect of postoperative ADT on continence recovery. (A) In the ADT subgroup, postoperative ADT did not affect continence recovery (Log rank p=0.232). (B) In the ADT +Docetaxel subgroup, postoperative ADT delayed continence recovery (Log rank p=0.005**). (C) In ADT+Abiraterone subgroup, postoperative ADT did not affect continence recovery (Log rank p=0.805).

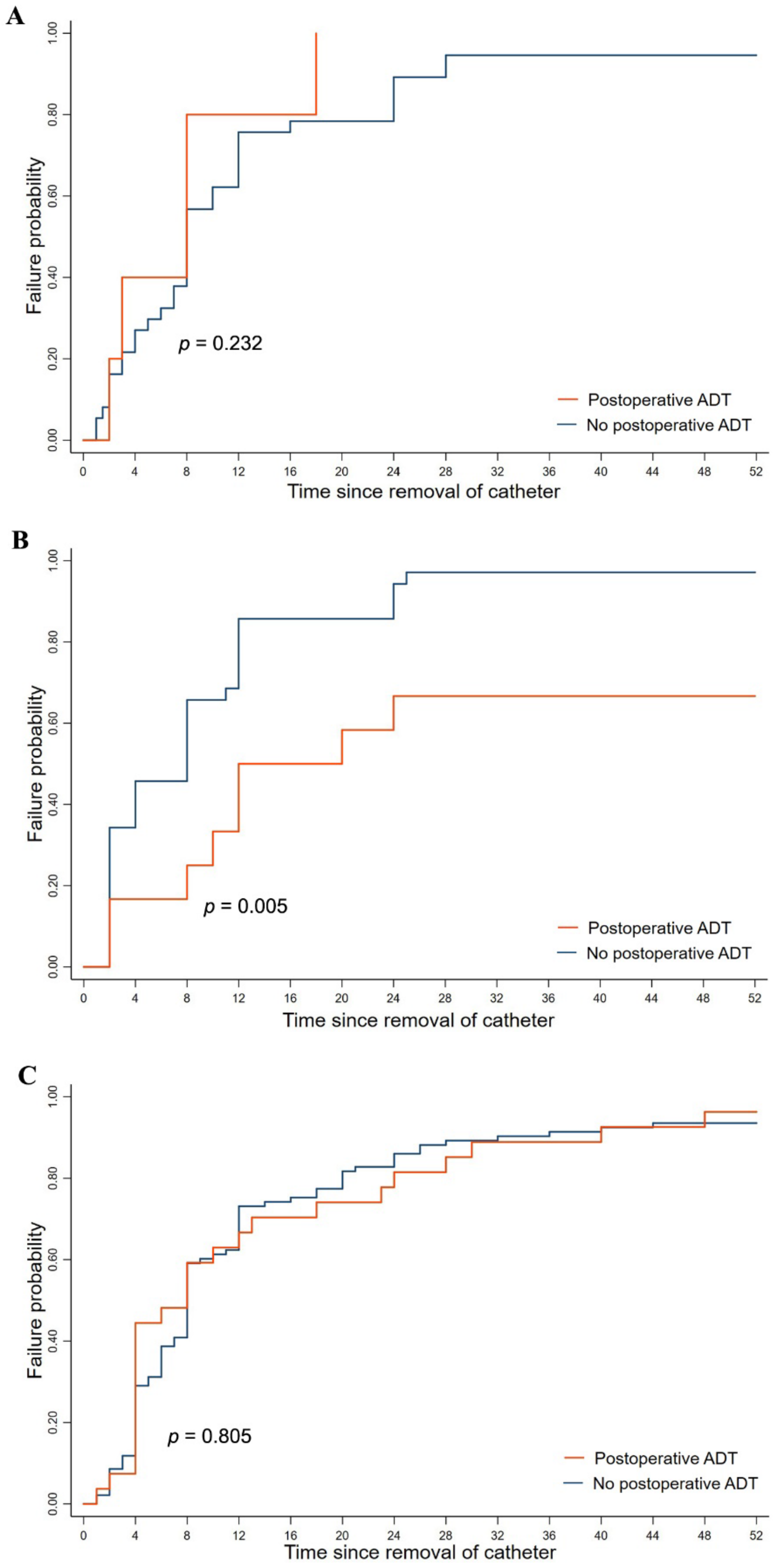

Supplement: Supplementary file 1 [file Image_1.pdf]
