# Supplementary material for: A retrospective study to evaluate the effect of preoperative hormonal therapy on continence recovery
Source: Front Oncol. 2023 Jan 13;12:1059410. doi: 10.3389/fonc.2022.1059410 (PMC9880985; doi:10.3389/fonc.2022.1059410)
Supplement: Supplementary file 2 [file Table_1.docx]

Supplementary Table 1. Logistic regression model for urinary continence

| **Factor** | **Univariate** |  | |  | **Multivariate** |  |
| --- | --- | --- | --- | --- | --- | --- |
|  | OR(95% CI) | *p* | |  | OR(95% CI) | *p* |
| **Immediate Urinary Continence** | | | | | | |
| Age | 0.97(0.90-1.04) | 0.343 |  | | - | - |
| BMI | 0.99(0.80-1.22) | 0.903 |  | | - | - |
| Initial T | 1.26(0.76-2.09) | 0.366 |  | | - | - |
| Apex invasion | 0.72(0.27-1.91) | 0.508 |  | | - | - |
| Preoperative PSA | 0.49(0.08-3.24) | 0.461 |  | | - | - |
| Preoperative volume | 1.05(0.99-1.10) | 0.067 |  | | - | - |
| Membranous urethral length | 1.15(0.87-1.52) | 0.339 |  | | - | - |
| Preoperative T | 0.39(0.19-0.78) | 0.007^**^ |  | | 0.46(0.24-0.85) | 0.014^*^ |
| Preoperative therapy |  |  |  | |  |  |
| ADT + Docetaxel vs ADT | 0.68(0.11-4.38) | 0.688 |  | | - | - |
| ADT + Abiraterone vs ADT | 1.15(0.27-5.02) | 0.848 |  | | - | - |
| Surgery approach | 1.21(0.44-3.31) | 0.718 |  | | - | - |
| Post-surgery ADT | 1.02(0.26-3.95) | 0.983 |  | | - | - |
|  |  |  |  | |  |  |
| **Urinary Continence at 1 month** | | | | | | |
| Age | 0.97(0.93-1.01) | 0.129 |  | | - | - |
| BMI | 1.06(0.95-1.18) | 0.334 |  | | - | - |
| Initial T | 0.85(0.60-1.22) | 0.383 |  | | - | - |
| Apex invasion | 0.91(0.52-1.60) | 0.736 |  | | - | - |
| Preoperative PSA | 1.01(0.92-1.10) | 0.917 |  | | - | - |
| Preoperative volume | 1.02(0.98-1.05) | 0.403 |  | | - | - |
| Membranous urethral length | 1.18(1.02-1.38) | 0.034^*^ |  | | 1.20(1.03-1.39) | 0.017^*^ |
| Preoperative T | 1.10(0.76-1.61) | 0.613 |  | | - | - |
| Preoperative therapy |  |  |  | |  |  |
| ADT + Docetaxel vs ADT | 1.15(0.46-2.87) | 0.733 |  | | - | - |
| ADT + Abiraterone vs ADT | 1.35(0.61-2.98) | 0.458 |  | | - | - |
| Surgery approach | 0.70(0.40-1.24) | 0.218 |  | | - | - |
| Post-surgery ADT | 1.04(0.51-2.12) | 0.906 |  | | - | - |
|  |  |  |  | |  |  |
| **Urinary Continence at 3 month** | | | | | | |
| Age | 0.96(0.91-1.01) | 0.144 |  | | - | - |
| BMI | 0.98(0.86-1.11) | 0.708 |  | | - | - |
| Initial T | 0.79(0.53-1.18) | 0.252 |  | | - | - |
| Apex invasion | 0.98(0.51-1.89) | 0.949 |  | | - | - |
| Preoperative PSA | 1.00(0.92-1.09) | 0.992 |  | | - | - |
| Preoperative volume | 0.99(0.95-1.03) | 0.627 |  | | - | - |
| Membranous urethral length | 1.32(1.10-1.59) | 0.003^**^ |  | | 1.27(1.07-1.51) | 0.006^**^ |
| Preoperative T | 1.09(0.71-1.66) | 0.691 |  | | - | - |
| Preoperative therapy |  |  |  | |  |  |
| ADT + Docetaxel vs ADT | 0.82(0.28-2.39) | 0.721 |  | | - | - |
| ADT + Abiraterone vs ADT | 0.71(0.29-1.76) | 0.458 |  | | - | - |
| Surgery approach | 1.48(0.76-2.87) | 0.252 |  | | - | - |
| Post-surgery ADT | 0.54(0.25-1.18) | 0.123 |  | | 0.50(0.24-1.02) | 0.056 |
|  | | | | | | |
| **Urinary Continence at 6 month** | | | | | | |
| Age | 1.00(0.93-1.06) | 0.889 |  | | - | - |
| BMI | 0.94(0.79-1.11) | 0.457 |  | | - | - |
| Initial T | 0.75(0.46-1.22) | 0.242 |  | | - | - |
| Apex invasion | 1.67(0.70-3.97) | 0.245 |  | | - | - |
| Preoperative PSA | 0.96(0.88-1.06) | 0.446 |  | | - | - |
| Preoperative volume | 0.99(0.93-1.04) | 0.605 |  | | - | - |
| Membranous urethral length | 1.44(1.12-1.86) | 0.004^**^ |  | | 1.34(1.07-1.67) | 0.011^*^ |
| Preoperative T | 1.68(0.97-2.93) | 0.066 |  | | - | - |
| Preoperative therapy |  |  |  | |  |  |
| ADT + Docetaxel vs ADT | 0.65(0.16-2.74) | 0.560 |  | | - | - |
| ADT + Abiraterone vs ADT | 0.57(0.16-2.02) | 0.387 |  | | - | - |
| Surgery approach | 1.51(0.62-3.63) | 0.363 |  | | - | - |
| Post-surgery ADT | 0.41(0.15-1.12) | 0.081 |  | | 0.46(0.19-1.12) | 0.087 |
|  | | | | | | |
| **Urinary Continence at 12 month** | | | | | | |
| Age | 1.02(0.94-1.11) | 0.677 |  | | - | - |
| BMI | 0.95(0.78-1.16) | 0.641 |  | | - | - |
| Initial T | 0.58(0.30-1.13) | 0.110 |  | | - | - |
| Apex invasion | 1.17(0.34-3.99) | 0.805 |  | | - | - |
| Preoperative PSA | 1.11(0.50-2.46) | 0.792 |  | | - | - |
| Preoperative volume | 1.01(0.93-1.10) | 0.799 |  | | - | - |
| Membranous urethral length | 1.42(1.01-1.99) | 0.042^*^ |  | | 1.36(1.01-1.84) | 0.044^*^ |
| Preoperative T | 1.68(0.82-3.44) | 0.153 |  | | - | - |
| Preoperative therapy |  |  |  | |  |  |
| ADT + Docetaxel vs ADT | 0.51(0.08-3.13) | 0.468 |  | | - | - |
| ADT + Abiraterone vs ADT | 1.14(0.20-6.63) | 0.883 |  | | - | - |
| Surgery approach | 2.11(0.64-6.99) | 0.221 |  | | - | - |
| Post-surgery ADT | 0.37(0.10-1.34) | 0.129 |  | | - | - |

^*^ *p* < 0.05 ; ^**^*p* < 0.01

Supplementary Table 2. Information on continuing ADT after RARP

|  | Postoperative ADT, n(%) | No postoperative ADT, n(%) |
| --- | --- | --- |
| Localized PCa | 2 (5.6) | 34 (94.4) |
| Locally advanced PCa | 28 (16.7) | 140 (83.3) |
| Oligometastatic PCa | 17 (65.4) | 9 (34.6) |
